# Prognostic Value of Long Noncoding RNAs in Patients with Gastrointestinal Cancer: A Systematic Review and Meta-Analysis

**DOI:** 10.1155/2018/5340894

**Published:** 2018-11-26

**Authors:** Weibiao Kang, Qiang Zheng, Jun Lei, Changyu Chen, Changjun Yu

**Affiliations:** ^1^Department of Gastrointestinal Surgery, Department of General Surgery, First Affiliated Hospital of Anhui Medical University, Hefei, China; ^2^Department of General Surgery, Lu'an People's Hospital, Luan, China; ^3^Department of General Surgery, First Affiliated Hospital of Anhui Traditional Medical University, Hefei, China

## Abstract

Gastrointestinal cancers (GICs) are a huge threat to human health, which mainly include esophageal, gastric, and colorectal cancers. The purpose of this study was to clarify the prognostic value of long noncoding RNAs (lncRNAs) in GICs. A total of 111 articles were included, and 13103 patients (3123 with esophageal cancer, 4972 with gastric cancer, and 5008 with colorectal cancer) were enrolled in this study. The pooled hazard ratio (HR) values and corresponding 95% confidence interval (95% CI) of overall survival (OS) related to different lncRNA expressions in esophageal, gastric, colorectal, and gastrointestinal cancer patients were 1.92 (1.70–2.16), 1.96 (1.77–2.16), 2.10 (1.87–2.36), and 2.00 (1.87–2.13), respectively. We have identified 74 lncRNAs which were associated closely with poor prognosis of GIC patients, including 58 significantly upregulated lncRNA expression and 16 significantly downregulated lncRNA expression. In addition, 47 of the included studies revealed relative mechanisms and 12 of them investigated the correlation between lncRNAs and microRNAs. Taken together, this meta-analysis supports that specific lncRNAs are significantly related to the prognosis of GIC patients and may serve as novel markers for predicting the prognosis of GIC patients. Furthermore, lncRNAs may have a promising contribution to lncRNA-based targeted therapy and clinical decision-making in the future.

## 1. Introduction

Gastrointestinal cancers (GICs) are one of the most common causes of cancer-related deaths with a high mortality worldwide, which mainly include esophageal, gastric, and colorectal cancers (EC, GC, and CRC). In addition to aging and expansion of world population, cancer-causing behaviors play a key role in the increasing largely global burden of GIC, such as smoking and changes in dietary patterns [1]. There are many therapy strategies applicable to GIC patients, such as surgery, neoadjuvant chemoradiotherapy, and adjuvant chemoradiotherapy [2], and GIC patients at early stage could be curable by receiving suitable treatment with a 90% five-year overall survival, However, five-year overall survivals are still poor for patients with advanced stages [3, 4]. Consequently, early diagnosis and selection of high-risk individuals with poor prognosis are important in the recovery of patients. However, effective methods to evaluate prognosis of GIC patients are still lacking nowadays. Currently, mounting reports have reported that noncoding RNA could be used to predict the prognosis of GIC patients, For example, microRNAs are potentially eligible for predicting the survival of GIC patients [5]. Many studies indicated that long noncoding RNAs (lncRNAs) could competitively suppress microRNAs by acting as molecular sponges recently [6]. Besides, aberrant expression of specific lncRNAs as molecular biomarkers was associated closely with prognosis of GIC patients and involved in targeted therapy, which might promote the development of novel prevention strategies and advanced therapies [7–12].

lncRNA is a long (more than 200 nucleotides) class of noncoding RNA that is often expressed in a disease-, tissue-, or stage-specific manner [13]. According to recent estimate, more than 28000 distinct lncRNAs are encoded by human genome and they regulate gene expression by means of different mechanisms, including chromatin modification, transcription, and posttranscriptional processing, which are becoming attractive therapeutic targets of cancers [14, 15]. Such upregulated lncRNA HOXA11-AS expression promotes tumor proliferation and invasion by scaffolding the chromatin modification factors PRC2, LSD1, and DNMT1 [16]. lncRNA FEZF1-AS1 recruits and bounds to LSD1 to epigenetically repress downstream gene p21, thereby promoting proliferation [17], and lncRNA GHET1 promotes gastric carcinoma cell proliferation by increasing c-Myc mRNA stability [18]. Furthermore, lncRNA plays crucial roles in the diverse biological processes such as development, differentiation, and carcinogenesis [19]. In addition, lncRNA may induce resistance of an anticancer drug. For example, upregulated lncRNA MALAT1 induces chemoresistance of CRC cells [20].

Recently, mounting evidences have indicated that various lncRNAs can function as oncogenes or tumor suppressor genes and the dysregulation of lncRNA expression as molecular biomarkers presented promising huge prognostic values in GIC patients [21–26]. However, the ability of evaluating relationship between multiple lncRNA expression and prognosis of GIC patients was limited due to monocentric, small samples and various experimental methods and criteria from different research departments. Therefore, the purpose of the study was to elaborate the relationship between multiple lncRNA expression and prognosis of GIC patients so that further understanding of prognostic values of lncRNAs might promote lncRNA-based target therapeutic development and make a clinical decision that is suitable for the individual quickly.

## 2. Materials and Methods

### 2.1. Search Strategy

To obtain the relevant studies for this meta-analysis, two authors (Weibiao Kang and Qiang Zheng) searched a wide range of database (PubMed, Web of Science, and Embase) independently up to August 27, 2018. Search terms are as follows: “LncRNA”, “Long non-coding RNAs”, “lncRNAs”, “lncRNA”, “Long ncRNA”, “LincRNAs”, “LINC RNA”, “Long ncRNAs”, “cancer”, “tumor”, “malignancy”, “carcinoma”, “neoplasia”, “neoplasm”, “gastrointestine”, “gastroenteric”, “colon”, “colorectal”, “rectum”, “intestinal”, “gastric”, “esophageal”, “esophagus”, “follow up studies”, “prognosis”, “prediction”, “survival”, “hazard ratio”, “incidence”, and “mortality”, which were combined with AND/OR.

### 2.2. Selection Criteria

All eligible studies were assessed and extracted data by the same two investigators independently based on the selection criteria. Inclusion criteria are the following: (1) patients who were diagnosed as having gastrointestinal cancer by pathologists and did not receive any preoperative chemotherapy or radiotherapy before obtaining samples; (2) predicting prognosis of full stage (I–IV) patients on the basis of the expression levels of lncRNAs; (3) the expression levels of lncRNAs were divided into high and low levels; (4) we could obtain overall survival (OS), disease-free survival (DFS), hazard ratio (HR), and 95% confidence interval (95% CI) directly from full text or extract survival data from Kaplan-Meier survival curves. Exclusion criteria are the following: (1) reviews, letters, case reports, statements, and not clinical related studies were excluded; (2) besides non-English and nonhuman studies, articles lack of data were also excluded; (3) studies focused on lncRNA variants or relationship between lncRNA expression and prognosis in different histological types of GIC. We resolved disagreements by discussing with the third investigator (Changjun Yu) and got consensus finally.

### 2.3. Data Extraction and Quality Assessment

The two authors (Weibiao Kang and Qiang Zheng) extracted data independently and got consensus finally. The characteristics collected of individual articles were as follows: author, year of publication, nation of population enrolled, number of patients, HR and 95% CI (OS/DFS), cut-off value, method, sample type, and follow-up. We assessed the quality of each study by using the guidelines for meta-analysis of observation studies in epidemiology (MOOSE) [27].

### 2.4. Statistical Analysis

Statistical analysis was conducted by Review Manager 5.2 (provided by Cochrane collaboration). *P* < 0.01 was considered statistically significant. The heterogeneity among studies was calculated by *Q* and *I*^2^ tests. *P* > 0.10 in combination with *I*^2^ < 50% indicated low heterogeneity; fixed-effect models should be used. Otherwise, random-effect model would be used finally. For some studies from which we could not extract HR and corresponding 95% CI (OS/DFS) directly, Engauge Digitizer 4.1 software was applied to obtain the necessary points and the relevant data from Kaplan-Meier survival curves, then HR and corresponding 95% CI were calculated by published methods proposed by Tierney et al. [28]. Additionally, forest plots of the pooled HR values and funnel plots used to analyse qualitatively publication bias were presented. Furthermore, we also applied sensitivity analysis for this meta-analysis.

## 3. Results

### 3.1. Study Identification and Characteristics

**Figure 1:**
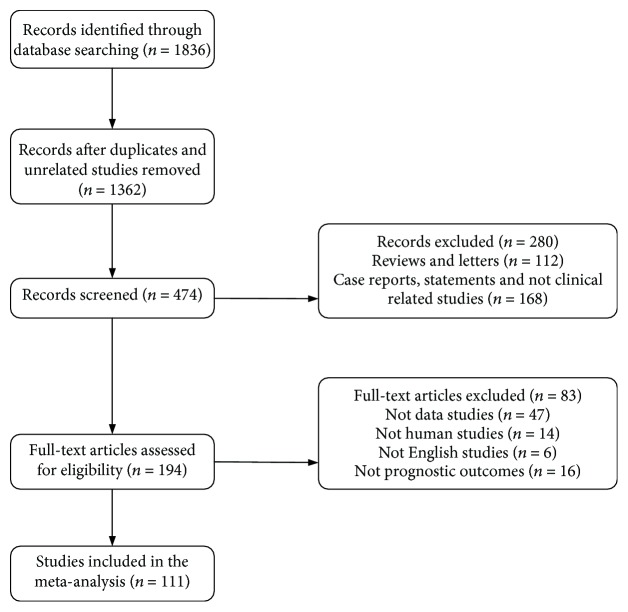
Study flow diagram.

According to the selection criteria, a total of 111 articles (21 EC, 47 GC, and 44 CRC; one study involved GC and CRC) involving 13103 patients (3123 with EC, 4972 with GC, and 5008 with CRC) were identified and included in the meta-analysis; specific steps were showed in Figure 1 [10–13, 15–26, 29–123]. Most of the studies taken into account refer to Asian population, especially china. Cut-off values of high or low lncRNA expression were mostly median or mean. Detection methods of lncRNA expression were mainly RT-PCR (reverse transcription PCR) or ISH (in situ hybridization). Sample types were almost from tissues. As for clinical outcome indicators, 74 studies [10–13, 16, 18–23, 25, 26, 29, 31–33, 36, 38, 40, 41, 43–47, 50, 51, 53–58, 61, 63, 64, 66–68, 71–74, 77, 78, 83, 85, 86, 88, 89, 91, 92, 96–102, 105–107, 109–112, 115–119, 121, 122] included overall survival (OS), 8 studies [17, 24, 30, 34, 79, 95, 114, 123] included disease-free survival (DFS), and another 29 studies [15, 35, 37, 39, 42, 48, 49, 52, 59, 60, 62, 65, 69, 70, 75, 76, 80–82, 84, 87, 90, 93, 94, 103, 104, 108, 113, 120] included both OS and DFS. We have identified 74 lncRNAs which were associated closely with poor prognosis of GIC patients, including 58 significantly upregulated lncRNA expression and 16 significantly downregulated lncRNA expression (Tables 1 and 2). Moreover, 47 of the included studies revealed relative mechanisms, and 12 of them investigated the correlation between lncRNAs and microRNAs (Table 3).

### 3.2. Meta-Analysis Findings

**Figure 2:**
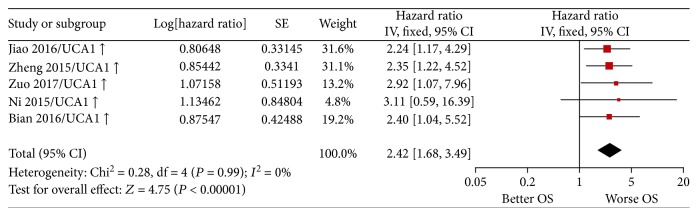
Forest plot showing the pooled HR and corresponding 95% CI of OS related to the expression level of lncRNA UCA1 in gastrointestinal cancer patients. HR: hazard ratio; CI: confidence interval; OS: overall survival.

**Figure 3:**
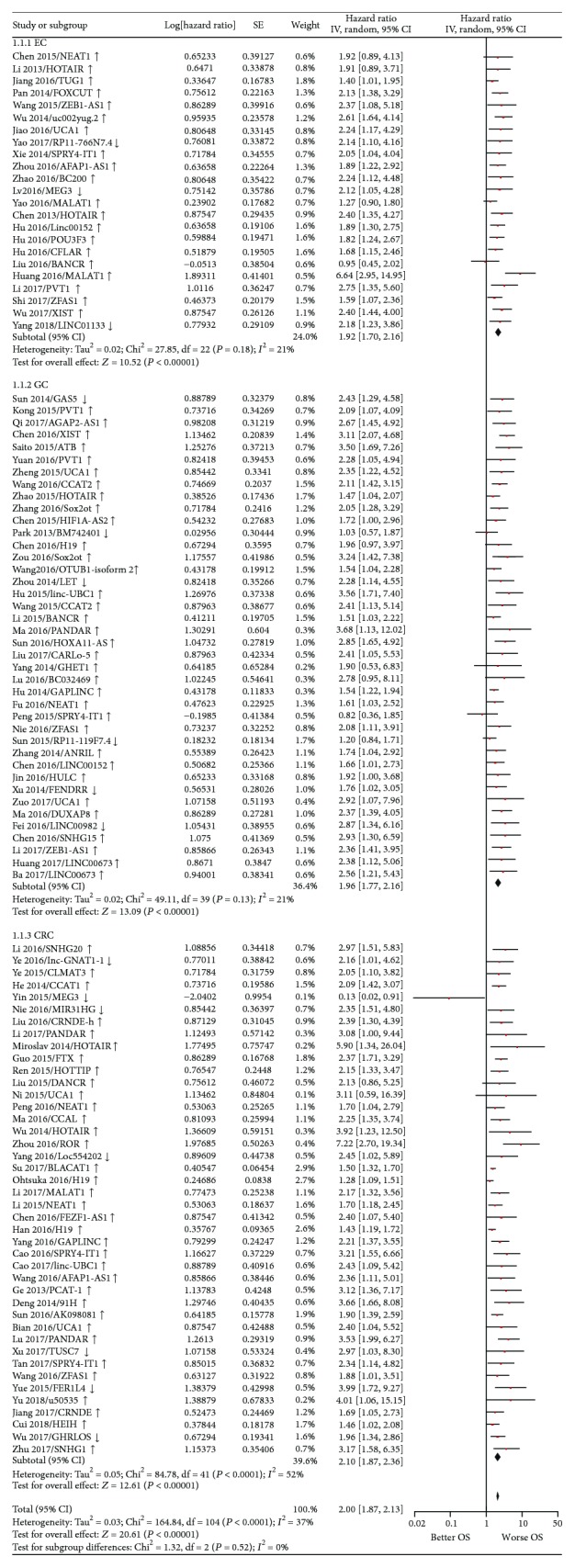
Forest plot showing the pooled HR (95% CI) of OS related to the expression level of different lncRNAs in gastrointestinal cancer patients. (1.1.1) Specific lncRNA expression in EC (esophageal cancer); (1.1.2) specific lncRNA expression in GC (gastric cancer); (1.1.3) specific lncRNA expression in CRC (colorectal cancer). HR: hazard ratio; CI: confidence interval; OS: overall survival.

**Figure 4:**
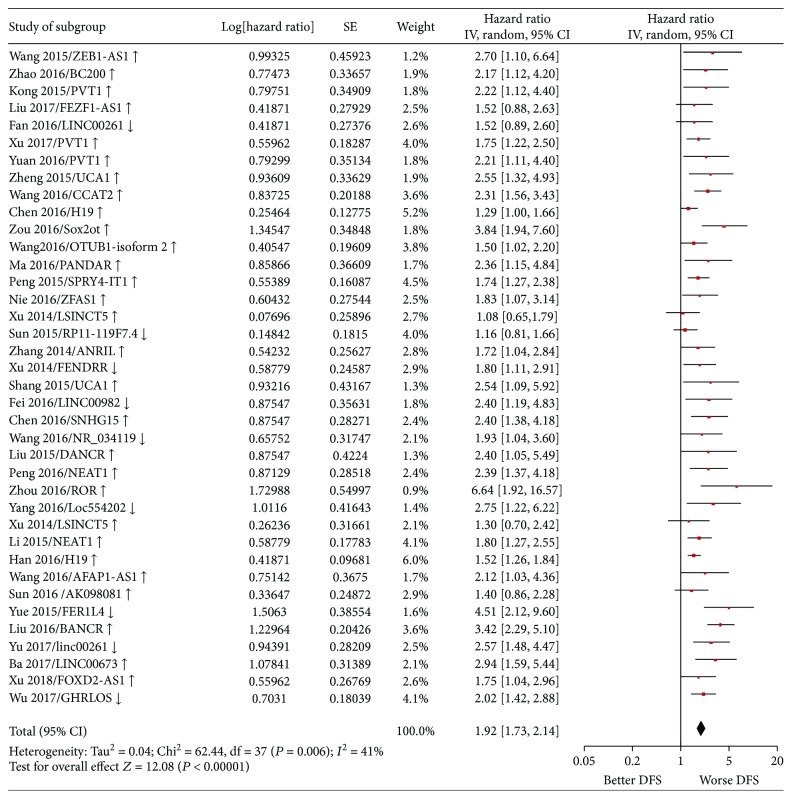
Forest plot showing the pooled HR (95% CI) of DFS related to the expression level of different lncRNAs in GIC patients. HR: hazard ratio; CI: confidence interval; DFS: disease-free survival; GIC: gastrointestinal cancer.

**Figure 5:**
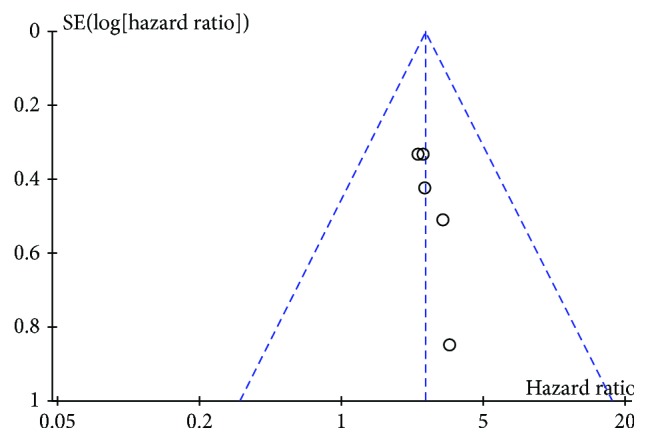
Funnel plot of included studies: highly expressed lncRNA UCA1 related to overall survival in gastrointestinal cancer patients.

**Figure 6:**
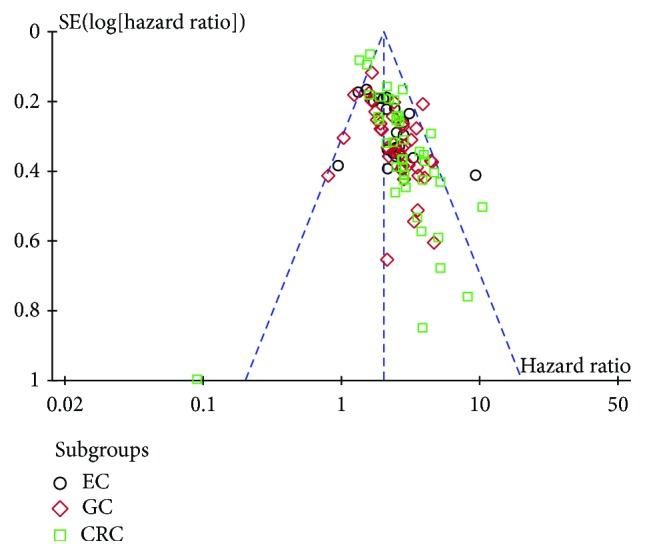
Funnel plot of included studies: aberrantly expressed lncRNAs related to overall survival in gastrointestinal cancer patients. EC: esophageal cancer; GC: gastric cancer; CRC: colorectal cancer.

**Figure 7:**
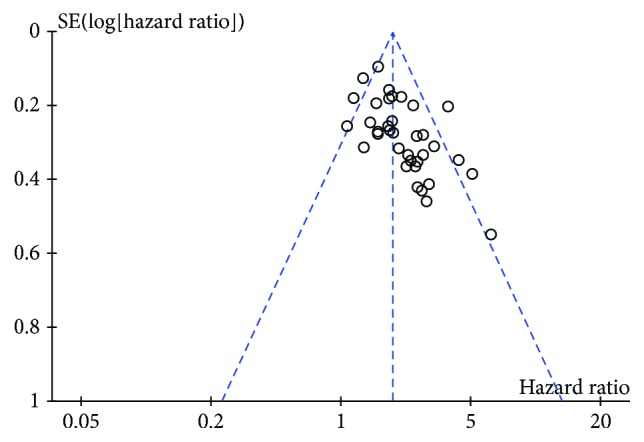
Funnel plot of included studies: aberrantly expressed lncRNAs related to disease-free survival in gastrointestinal cancer patients.

Random-effect and fixed-effect models were applied to evaluate the pooled hazard ratio (HR) and its corresponding 95% confidence interval (CI) of OS or DFS based on the heterogeneity level. The pooled HR value (95% CI) of OS which correlated with the expression of lncRNA-UCA1 [37, 64, 66, 96, 97] was 2.42 (1.68–3.49) with low heterogeneity (*P* = 0.99, *I*^2^ = 0%) and statistically significant (*P* < 0.00001) (Figure 2). For all included studies, the pooled HR values (95% CI) of OS related to different lncRNA expressions in EC, GC, and CRC patients were 1.92 (1.70–2.16), 1.96 (1.77–2.16), and 2.10 (1.87–2.36), respectively. And the pooled HR value (95% CI) of OS related to different lncRNA expressions was 2.00 (1.87–2.13) in GIC with moderate heterogeneity (*P* = 0.0001, *I*^2^ = 37%) and statistically significant (*P* < 0.00001) (Figure 3). Besides, the pooled HR value (95% CI) of DFS related to different lncRNA expressions was 1.92 (1.73–2.14) in GIC patients with moderate heterogeneity (*P* = 0.006, *I*^2^ = 41%) and statistically significant (*P* < 0.00001) (Figure 4). Furthermore, funnel plots of included studies related to lncRNA-UCA1, OS, and DFS in GIC patients were presented in Figures 5, 6, and 7, respectively. These figures are approximately symmetrical, and we can think that there is no obvious publication bias.

## 4. Discussion

GIC is still a huge threat to human health in spite of ongoing emergence of new anticancer drugs because of chemotherapy resistance and metastasis inducing poor prognosis. In the last decade, more and more studies focused on the clinical roles of lncRNAs and many reports indicated that lncRNA can be a molecular biomarker in gastrointestinal cancer patients for predicting prognosis. However, the prognostic value of lncRNAs that need to be clarified, verified, and summarized was limited by various research centers and small samples.

The purpose of this study was to elucidate the relationship between multiple lncRNA expressions and prognosis of GIC patients. Through big data meta-analysis, we provided evidence to illustrate the prognostic value of aberrantly expressed lncRNAs in GIC patients. The results from this meta-analysis showed that the pooled HR values (95% CI) of OS and DFS related to different lncRNA expressions in GIC patients were 2.00 (1.87–2.13) and 1.92 (1.73–2.14), respectively, which implied that aberrantly expressed lncRNAs may serve as cancer biomarkers in GIC patients. By detecting expression levels of specific lncRNAs in tissue or other body fluids, we cannot only make appropriate clinical decisions based on different prognoses but also monitor the therapeutic efficacy of GIC patients receiving different treatments. In addition, lncRNAs may be used to screen patients at high risk at the early stage based on abnormal expression. Moreover, elevated lncRNA-UCA1 expression promoted tumor cell migration, invasion, EMT, proliferation, and chemoresistance and inhibited its apoptosis by different target genes, which was associated with poor prognosis. For example, Jiao et al. [66] reported that lncRNA-UCA1 as a competing endogenous RNA (ceRNA) of Sox4 enhanced tumor cell proliferation by targeting miR-204 and Sox4 and Bian et al. [96] demonstrated that lncRNA-UCA1 promoted tumor cell proliferation and 5-fluorouracil resistance by functioning as a ceRNA of miR-204-5p. The pooled HR value (95% CI) of OS which correlated with the expression of lncRNA-UCA1 was 2.42 (1.68–3.49) with low heterogeneity (*P* = 0.99, *I*^2^ = 0%) and statistically significant (*P* < 0.00001). Therefore, lncRNA-UCA1 as a molecular biomarker can be applied in predicting the prognosis of GIC patients. Generally, predicting prognosis of patients and exploring mechanisms of lncRNAs play pivotal roles in clinical decision-making and development of novel targeted gene therapies. Therefore, we summarized the researches involved in mechanisms of lncRNAs; we found that 37 lncRNAs had explicit targets and 11 lncRNAs as ceRNAs regulated cancer progression by sponging corresponding microRNAs. These studies demonstrated that the potential relationship between lncRNAs and microRNAs plays a key role in tumor pathogenesis and promoted carcinogenic study and development of gene therapy. Many studies focusing on the same lncRNA revealed different targets, and the underlying correlation between lncRNAs and microRNAs was still unclear. In the future, we should focus on the interrelationship between lncRNA and microRNA or other types of RNA, in achieving targeted treatment by simultaneous intervention of multiple types of RNA.

Several limitations should not be ignored. First, most of included patients were from East Asia, especially China, which makes our conclusions may just be suitable for Chinese patients. Second, the cut-off values and detection methods in evaluating different lncRNA expressions were various in different included studies, which may lead to heterogeneity between studies. Third, language bias was also one of the limitations, because we only enrolled English papers in the meta-analysis. Fourth, the majority of authors were generally more inclined to report positive results so that the pooled effect values calculated might overestimated the predictive significance of lncRNAs in prognosis of GIC patients; the publication bias have reached a consensus. Fifth, we calculated the HR estimates from the Kaplan-Meier survival curves because of some studies from which we could not extract HR and 95% CI directly. Sixth, the confounding factors in some included studies without the adjusted HR values would lead to high heterogeneity.

In summary, this meta-analysis supports the fact that specific lncRNAs are significantly related to the prognosis of GIC patients and may serve as novel markers for predicting the prognosis in GIC patients. In addition, lncRNAs may have a promising contribution to lncRNA-based targeted therapy and clinical decision-making in the future.

## Figures and Tables

**Table 1:** Characteristics of studies and lncRNA expression related to OS in GIC patients.

References	lncRNAs (*n* = 105)	Year	Nations	Number (*n* = 12178)	OS		Cut-off value	Detection methods	Sample types	Follow-up
					HR	95% CI				
Sun et al. [13]	RNAGAS5^↓^	2014	China	89 GC	2.43^∗^	1.29–4.59	Median	RT-PCR	Tissue	<40
Li et al. [29]	SNHG20^↑^	2016	China	107 CRC	2.97^∗^	1.51–5.82	YI	RT-PCR	Tissue	<40
Kong et al. [15]^!^	PVT1^↑^	2015	China	80 GC	2.09^∗^	1.07–4.10	Median	RT-PCR	Tissue	<40
Qi et al. [31]	AGAP2-AS1^↑^	2017	China	50 GC	2.67^#^	1.45–4.93	Median	RT-PCR	Tissue	6–36^#^
Chen et al. [32]	XIST^↑^	2016	China	106 GC	3.11	1.67–3.78	Median	RT-PCR	Tissue	<120
Ye et al. [33]	lnc-GNAT1-1^↓^	2016	China	68 CRC	2.16^∗^	1.01–4.63	Median	RT-PCR	Tissue	<20
Saito et al. [21]	ATB^↑^	2015	Japan	183 GC	3.50^∗^	1.73–7.44	Median	RT-PCR	Tissue	0.192–134.4
Yuan et al. [35]^!^	PVT1^↑^	2016	China	111 GC	2.28^∗^	1.05–4.93	Median	RT-PCR	Tissue	20–48
Ye et al. [36]	CLMAT3^↑^	2015	China	90 CRC	2.05^∗^	1.10–3.82	Dichotomize	RT-PCR	Tissue	<45
Zheng et al. [37]^!^	UCA1^↑^	2015	China	112 GC	2.35^∗^	1.22–4.52	Dichotomize	RT-PCR	Tissue	<92
Chen et al. [38]	NEAT1^↑^	2015	China	96 EC	1.92^∗^	1.40–6.49	YI	RT-PCR	Tissue	<80
Wang et al. [39]^!^	CCAT2^↑^	2016	China	108 GC	2.11^∗^	1.44–3.20	Median	RT-PCR	Tissue	<70
Zhao et al. [22]	HOTAIR^↑^	2015	China	168 GC	1.47^∗^	1.04–2.06	Median	RT-PCR	Tissue	<70
Zhang et al. [40]	Sox2ot^↑^	2016	China	132 GC	2.05^∗^	1.28–3.30	Median	RT-PCR	Tissue	<96
Chen et al. [41]	HIF1A-AS2^↑^	2015	China	83 GC	1.72^∗^	1.00–2.96	Median	RT-PCR	Tissue	<60
Li et al. [10]	HOTAIR^↑^	2013	China	100 EC	1.91	1.06–4.00	125-fold	RT-PCR	Tissue	<60
Yue et al. [42]^!^	FER1L4^↓^	2015	China	70 CC	3.99^∗^	1.67–9.01	Median	RT-PCR	Tissue	<80
He et al. [43]	CCAT1^↑^	2014	China	48 CC	2.09^#^	1.42–3.06	Median	RT-PCR	Tissue	24–37^#^
Yin et al. [44]	MEG3^↓^	2015	China	62 CRC	0.13^∗^	0.02–0.99	Mean	RT-PCR	Tissue	<60
Nie et al. [45]	MIR31HG^↓^	2016	China	48 CC	2.35^#^	1.15–4.79	Median	RT-PCR	Tissue	3–36^#^
Park et al. [46]	BM742401^↓^	2013	Korea	113 GC	1.03^∗^	0.57–1.88	Median	RT-PCR	Tissue	<80
Liu et al. [23]	CRNDE-h^↑^	2016	China	148 CRC	2.39^∗^	1.30–4.39	Median	RT-PCR	Serum	1–65
Li et al. [47]	PANDAR^↑^	2017	China	102 CRC	3.08^∗^	0.84–7.89	Median	RT-PCR	Tissue	<60
Chen et al. [48]^!^	H19^↑^	2016	China	128 GC	1.96^∗^	0.97–3.97	Median	RT-PCR	Tissue	20–48
Zou et al. [49]^!^	Sox2ot^↑^	2016	China	155 GC	3.24^∗^	1.24–6.43	Median	RT-PCR	Tissue	<70
Jiang et al. [50]	TUG1^↑^	2016	China	218 EC	1.40^∗^	1.01–1.95	NR	RT-PCR	Tissue	12–72
Svoboda et al. [51]	HOTAIR^↑^	2014	Czech	84 CRC	5.9	1.34–26.1	Median	RT-PCR	Blood	12–54
Wang et al. [52]^!^	OTUB1-isoform 2^↑^	2016	China	156 GC	1.54	1.04–2.27	Median	RT-PCR	Tissue	<80
Guo et al. [53]	FTX^↑^	2015	China	187 CRC	2.37	1.42–2.74	Median	RT-PCR	Tissue	<60
Pan et al. [54]	FOXCUT^↑^	2014	China	82 EC	2.13^#^	1.38–3.29	Mean	RT-PCR	Tissue	1–72
Zhou et al. [55]	LET^↓^	2014	China	93 GC	2.28	1.30–5.18	Mean	RT-PCR	Tissue	<60
Hu et al. [56]	linc-UBC1^↑^	2015	China	85 GC	3.56^#^	1.71–7.39	Median	RT-PCR	Tissue	<100
Wang et al. [57]	CCAT2^↑^	2015	China	86 GC	2.41	1.19–5.42	Mean	RT-PCR	Tissue	<60
Ren et al. [58]	HOTTIP^↑^	2015	China	156 CRC	2.15	1.31–3.42	Median	RT-PCR	Tissue	33–65
Liu et al. [59]^!^	DANCR^↑^	2015	China	104 CRC	2.13^∗^	1.16–7.06	Median	RT-PCR	Tissue	<60
Wang et al. [60]^!^	ZEB1-AS1^↑^	2015	China	87 EC	2.37	1.28–6.12	Median	RT-PCR	Tissue	<61
Li et al. [61]	BANCR^↑^	2015	China	184 GC	1.51^∗^	1.03–2.23	Median	RT-PCR	Tissue	5–93
Ma [62]^!^	PANDAR^↑^	2016	China	100 GC	3.68	1.13–12.06	NR	RT-PCR	Tissue	2–36
Huang et al. [63]	MALAT1^↑^	2016	China	132 EC	6.64	2.95–14.95	NR	RT-PCR	Tissue	<60
Ni et al. [64]	UCA1^↑^	2015	China	54 CRC	3.11^#^	0.59–16.39	Median	RT-PCR	Tissue	9–51^#^
Wu et al. [25]	uc002yug.2^↑^	2014	China	684 EC	2.61	1.50–3.78	NR	RT-PCR	Tissue	<140
Sun et al. [16]	HOXA11-AS^↑^	2016	China	85 GC	2.85^#^	1.65–4.91	Median	ISH	Tissue	9–36
Peng et al. [65]^!^	NEAT1^↑^	2016	China	56 CRC	1.70^#^	1.04–2.80	NR	RT-PCR	Tissue	<60
Jiao et al. [66]	UCA1^↑^	2016	China	66 EC	2.24^#^	1.17–4.29	Median	RT-PCR	Tissue	5–30^#^
Liu and Shangguan [67]	CARLo-5^↑^	2017	China	240 GC	2.41^∗^	1.13–5.94	0.041	RT-PCR	Tissue	<60
Ma et al. [11]	CCAL^↑^	2016	China	252 CRC	2.25^∗^	1.35–3.74	Median	RT-PCR	Tissue	<100
Yang et al. [18]	GHET1^↑^	2014	China	42 GC	1.90^#^	0.53–6.85	Median	RT-PCR	Tissue	7–40^#^
Wu et al. [68]	HOTAIR^↑^	2014	China	120 CC	3.92	1.23–12.50	5-fold	RT-PCR	Tissue	10–72
Zhou et al. [69]^!^	ROR^↑^	2016	China	60 CC	7.22^∗^	2.43–17.43	Median	RT-PCR	Tissue	<80
Yang et al. [70]^!^	Loc554202^↓^	2016	China	178 CRC	2.45	1.34–7.74	Median	RT-PCR	Tissue	<70
Lü et al. [71]	BC032469^↑^	2016	China	58 GC	2.78^#^	0.95–8.09	Mean	RT-PCR	Tissue	<23
Su et al. [72]	BLACAT1^↑^	2017	China	48 CRC	1.50^∗^	1.32–1.70	Mean	RT-PCR	Tissue	<60
Hu et al. [12]	GAPLINC^↑^	2014	China	90 GC	1.54^∗^	1.22–1.94	Median	ISH	Tissue	<80
Fu et al. [73]	NEAT1^↑^	2016	China	140 GC	1.61	1.03–2.53	Median	RT-PCR	Tissue	<96
Yao et al. [26]	RP11-766N7.4^↓^	2017	China	50 EC	2.14^#^	1.10–4.15	Median	RT-PCR	Tissue	32–60^#^
Xie et al. [74]	SPRY4-IT1^↑^	2014	China	92 EC	2.05	1.04–4.03	Median	RT-PCR	Tissue	3–60
Peng [75]^!^	SPRY4-IT1^↑^	2015	China	175 GC	0.82^∗^	0.31–1.57	Median	RT-PCR	Tissue	<60
Nie et al. [76]^!^	ZFAS1^↑^	2016	China	54 GC	2.08^#^	1.11–3.93	Median	RT-PCR	Tissue	3–36^#^
Ohtsuka et al. [77]	H19^↑^	2016	USA	117 CC	1.28^∗^	1.08–1.50	0.64	RT-PCR	Tissue	<90
Li et al. [20]	MALAT1^↑^	2017	China	68 CRC	2.17^#^	1.32–3.55	Median	RT-PCR	Tissue	1–51^#^
Zhou et al. [78]	AFAP1-AS1^↑^	2016	China	162 EC	1.89^∗^	1.22–2.92	Median	RT-PCR	Tissue	6–72
Sun et al. [80]^!^	RP11-119F7.4^↓^	2015	China	96 GC	1.20^#^	0.84–1.71	Median	RT-PCR	Tissue	<100
Zhang et al. [81]^!^	ANRIL^↑^	2014	China	120 GC	1.74^∗^	1.04–2.93	3-fold	RT-PCR	Tissue	<60
Li et al. [82]^!^	NEAT1^↑^	2015	China	239 CRC	1.70^∗^	1.18–2.45	2-fold	RT-PCR	Tissue	<60
Chen et al. [83]	LINC00152^↑^	2016	China	97 GC	1.66^∗^	1.01-2.73	Median	RT-PCR	Tissue	<60
Chen et al. [19]	FEZF1-AS1^↑^	2016	China	153 CRC	2.40^∗^	1.07–5.41	NR	ISH	Tissue	<100
Han et al. [84]^!^	H19^↑^	2016	China	83 CRC	1.43^∗^	1.24–1.79	3-fold	RT-PCR	Tissue	<50
Yang et al. [85]	GAPLINC^↑^	2016	China	180 CRC	2.21^∗^	1.38–3.57	NR	ISH	Tissue	<100
Jin et al. [86]	HULC^↑^	2016	China	54 GC	1.92^#^	1.00–3.67	2-fold	RT-PCR	Serum	11–32^#^
Cao et al. [87]^!^	BC200^↑^	2016	China	70 EC	2.24^∗^	1.12–4.49	Median	RT-PCR	Tissue	<50
Cao et al. [88]	SPRY4-IT1^↑^	2016	China	84 CRC	3.21^∗^	1.55–6.67	2.87-fold	RT-PCR	Tissue	3–36
Gao et al. [89]	linc-UBC1^↑^	2017	China	96 CRC	2.43^∗^	1.09–5.42	Median	RT-PCR	Tissue	<60
Wang et al. [90]^!^	AFAP1-AS1^↑^	2016	China	52 CRC	2.36	1.11–5.01	Median	RT-PCR	Tissue	<50
Ge et al. [91]	PCAT-1^↑^	2013	China	108 CRC	3.12	1.36–7.19	NR	RT-PCR	Tissue	<100
Deng et al. [92]	91H^↑^	2014	China	72 CRC	3.66	1.66–8.10	2.86-fold	RT-PCR	Tissue	2–36
Sun et al. [93]^!^	AK098081^↑^	2016	China	84 CRC	1.90^∗^	1.39–2.58	Mean	RT-PCR	Tissue	1–118^#^
Xu et al. [94]^!^	FENDRR^↓^	2014	China	158 GC	1.76	1.04–3.12	Median	RT-PCR	Tissue	20–48
Bian et al. [96]	UCA1^↑^	2016	China	90 CRC	2.40^∗^	1.04-5.50	Median	RT-PCR	Tissue	<100
Zuo et al. [97]	UCA1^↑^	2017	China	37 GC	2.92^∗^	1.07–7.96	Median	RT-PCR	Tissue	<40
Lu et al. [98]	PANDAR^↑^	2017	China	124 CRC	3.53^∗^	1.41–4.45	Median	RT-PCR	Tissue	<60
Lv et al. [99]	MEG3^↓^	2016	China	96 EC	2.12	1.05–4.27	NR	RT-PCR	Tissue	<120
Xu et al. [100]	TUSC7^↓^	2017	China	63 CRC	2.92	1.03–8.33	NR	RT-PCR	Tissue	<120
Ma et al. [101]	DUXAP8^↑^	2016	China	72 GC	2.37^#^	1.39–4.05	Median	RT-PCR	Tissue	5–36^#^
Fei et al. [103]^!^	LINC00982^↓^	2016	China	106 GC	2.87^∗^	1.34–6.17	Median	RT-PCR	Tissue	20–48
Chen et al. [104]^!^	SNHG15^↑^	2016	China	106 GC	2.93^∗^	1.30–6.58	Median	RT-PCR	Tissue	20–48
Tan et al. [105]	SPRY4-IT1^↑^	2017	China	106 CRC	2.34^∗^	1.14–4.83	Mean	RT-PCR	Tissue	<70
Wang and Xing [106]	ZFAS1^↑^	2016	China	159 CRC	1.88^∗^	1.01–3.53	Median	RT-PCR	Tissue	<101
Yao et al. [107]	MALAT-1^↑^	2016	China	137 EC	1.27^#^	0.90–1.80	0.5-fold	RT-PCR	Tissue	3–36^#^
Liu et al. [108]^!^	BANCR^↑^	2016	China	142 EC	0.95^∗^	0.21–0.95	Median	RT-PCR	Tissue	1–60^#^
Chen et al. [109]	HOTAIR^↑^	2013	China	78 EC	2.40^∗^	1.35–4.28	Mean	RT-PCR	Tissue	2–60
Hu et al. [102]^a^	Linc00152^↑^	2016	China	205 EC	1.89	1.22–2.58	Upper 95% CI in control group	RT-PCR	Plasma	<60
	POU3F3^↑^				1.82	1.17–2.51				
	CFLAR^↑^				1.68	1.08–2.32				
Yu et al. [110]	u50535^↑^	2018	China	98CRC	4.01^∗^	1.06–15.14	NR	RT-PCR	Tissue	<60
Jiang et al. [111]	CRNDE^↑^	2017	China	251CRC	1.69^∗^	1.05–2.74	NR	ISH	Tissue	1–117
Cui et al. [112]	HEIH^↑^	2018	China	84CRC	1.46^∗^	1.02–2.08	Median	RT-PCR	Tissue	<60
Wu et al. [113]^!^	GHRLOS^↓^	2017	China	366CRC	1.96^∗^	1.34–2.86	1/2-fold	RT-PCR	Tissue	5–85
Li et al. [115]	ZEB1-AS1^↑^	2017	China	24GC	2.36^∗^	1.41–3.96	Median	RT-PCR	Tissue	72
Huang et al. [116]	LINC00673^↑^	2017	China	73GC	2.38^∗^	1.12–5.06	2-fold	RT-PCR	Tissue	<20
Li et al. [117]	PVT1^↑^	2017	China	104ESCC	2.75^∗^	1.35–5.59	Median	RT-PCR	Tissue	<80
Shi et al. [118]	ZFAS1^↑^	2017	China	246ESCC	1.59^∗^	1.07–2.36	Median	RT-PCR	Tissue	114
Wu et al. [119]	XIST^↑^	2017	China	127ESCC	2.4^∗^	1.44–4.01	Median	RT-PCR	Tissue	<80
Ba et al. [120]	LINC00673^↑^	2017	China	79GC	2.56^∗^	1.01–4.54	Median	RT-PCR	Tissue	<50
Zhu et al. [121]	SNHG1^↑^	2017	China	108CRC	3.17^∗^	1.55–6.21	Median	RT-PCR	Tissue	<50
Yang et al. [122]	LINC01133^↓^	2018	China	149ESCC	2.18^∗^	1.23–3.85	Median	RT-PCR	Tissue	<60

^a^One study involved lncRNA Linc00152, lncRNA POU3F3, and lncRNA CFLAR. ∗ indicates adjusted HR; # indicates calculated HR of OS and follow-up time; ! indicates studies included OS and DFS; ↑ or ↓ indicates upregulated or downregulated with poor prognosis. OS: overall survival; DFS: disease-free survival; HR: hazard ratio; CI: confidence interval; EC: esophageal cancer; GC: gastric cancer; CRC: colorectal cancer; GIC: gastrointestinal cancer; NR: no report; YI: Youden index; RT-PCR: reverse transcription PCR; ISH: in situ hybridization.

**Table 2:** Characteristics of studies and lncRNAs expression related to DFS in GIC patients.

References	lncRNAs (*n* = 37)	Year	Nations	Number (*n* = 4360)	DFS		Cut-off value	Detection methods	Sample types	Follow-up
					HR	95% CI				
Kong et al. [15]^!^	PVT1^↑^	2015	China	80GC	2.22^∗^	1.13–4.44	Median	RT-PCR	Tissue	<40
Liu et al. [17]	FEZF1-AS1^↑^	2017	China	82GC	1.52^#^	0.88–2.63	2-fold	RT-PCR	Tissue	1–43^#^
Fan et al. [30]	LINC00261^↓^	2016	China	138GC	1.81^∗^	1.06–3.10	Median	RT-PCR	Tissue	20–48
Xu et al. [34]	PVT1^↑^	2017	China	190GC	1.75	1.25–2.56	Mean	RT-PCR	Tissue	1–85
Yuan et al. [35]^!^	PVT1^↑^	2016	China	111GC	2.21^∗^	1.11–4.40	Median	RT-PCR	Tissue	20–48
Zheng et al. [37]^!^	UCA1^↑^	2015	China	112GC	2.55^∗^	1.33–4.97	Dichotomize	RT-PCR	Tissue	<92
Wang et al. [39]^!^	CCAT2^↑^	2016	China	108GC	2.31^∗^	1.55–3.42	Median	RT-PCR	Tissue	<70
Yue et al. [42]^!^	FER1L4^↓^	2015	China	70CC	4.51^∗^	1.99–9.02	Median	RT-PCR	Tissue	<80
Chen et al. [48]^!^	H19^↑^	2016	China	128GC	1.29^∗^	1.00-1.65	Median	RT-PCR	Tissue	20–48
Zou et al. [49]^!^	Sox2ot^↑^	2016	China	155GC	3.84^∗^	1.87–7.33	Median	RT-PCR	Tissue	<70
Wang et al. [24]	NR_034119^↓^	2016	China	107CRC	1.93^∗^	1.04–3.61	NR	RT-PCR	Serum	11–74
Wang et al. [52]^!^	OTUB1-isoform 2^↑^	2016	China	156GC	1.50^∗^	1.02–2.20	Median	RT-PCR	Tissue	<80
Liu et al. [59]^!^	DANCR^↑^	2015	China	104CRC	2.40^∗^	1.39–7.28	Median	RT-PCR	Tissue	<60
Wang et al. [60]^!^	ZEB1-AS1^↑^	2015	China	87EC	2.7	1.38–8.35	Median	RT-PCR	Tissue	<61
Ma et al. [62]^!^	PANDAR^↑^	2016	China	100GC	2.36	1.15–4.83	NR	RT-PCR	Tissue	2–36
Peng et al. [65]^!^	NEAT1^↑^	2016	China	56CRC	2.39^#^	1.37–4.19	NR	RT-PCR	Tissue	<60
Zhou et al. [69]^!^	ROR^↑^	2016	China	60CC	5.64^∗^	1.92–16.58	Median	RT-PCR	Tissue	<80
Yang et al. [70]^!^	Loc554202^↓^	2016	China	178CRC	2.75	1.55–7.93	Median	RT-PCR	Tissue	<70
Peng et al. [75]^!^	SPRY4-IT1^↑^	2015	China	175GC	1.74^∗^	1.32–2.48	Median	RT-PCR	Tissue	<60
Nie et al. [76]^!^	ZFAS1^↑^	2016	China	54GC	1.83^#^	1.07–3.15	Median	RT-PCR	Tissue	3–36^#^
Xu et al. [79]^a^	LSINCT5^↑^	2014	China	71GC	1.08^∗^	1.29–3.56	Mean	RT-PCR	Tissue	<72
				74CRC	1.30^∗^	1.11–3.84	Mean	RT-PCR	Tissue	<72
Sun et al. [80]^!^	RP11-119F7.4^↓^	2015	China	96GC	1.16^#^	0.81–1.65	Median	RT-PCR	Tissue	<100
Zhang et al. [81]^!^	ANRIL^↑^	2014	China	120GC	1.72^∗^	1.04–2.84	3-fold	RT-PCR	Tissue	<60
Li et al. [82]^!^	NEAT1^↑^	2015	China	239CRC	1.80^∗^	1.27–2.55	2-fold	RT-PCR	Tissue	<60
Han et al. [84]^!^	H19^↑^	2016	China	83CRC	1.52^∗^	1.30–1.90	3-fold	RT-PCR	Tissue	<50
Cao et al. [87]^!^	BC200^↑^	2016	China	70EC	2.17^∗^	1.12–4.19	Median	RT-PCR	Tissue	<50
Wang et al. [90]^!^	AFAP1-AS1^↑^	2016	China	52CRC	2.12	1.03-4.35	Median	RT-PCR	Tissue	<50
Sun et al. [93]^!^	AK098081^↑^	2016	China	84CRC	1.40^#^	0.86–2.28	Mean	RT-PCR	Tissue	1–118^#^
Xu et al. [94]^!^	FENDRR^↓^	2014	China	158GC	1.8	1.11–2.91	Median	RT-PCR	Tissue	20–48
Shang et al. [95]	UCA1^↑^	2016	China	77GC	2.54	1.09–5.92	NR	RT-PCR	Tissue	<60
Fei et al. [103]^!^	LINC00982^↓^	2016	China	106GC	2.40^∗^	1.19--4.81	Median	RT-PCR	Tissue	20–48
Chen et al. [104]^!^	SNHG15^↑^	2016	China	106GC	2.40^∗^	1.38–4.18	Median	RT-PCR	Tissue	20–48
Liu et al. [108]^!^	BANCR^↑^	2016	China	142EC	3.42^#^	2.29–5.10	Median	RT-PCR	Tissue	1–60^#^
Wu et al. [113]^!^	GHRLOS^↓^	2017	China	366CRC	2.02^∗^	1.42–2.88	1/2-fold	RT-PCR	Tissue	5–85
Yu et al. [114]	linc00261^↓^	2017	China	80GC	2.57^∗^	1.39–4.20	NR	RT-PCR	Tissue	<30
Ba et al. [120]	LINC00673^↑^	2017	China	79GC	2.94^∗^	1.23–4.21	Median	RT-PCR	Tissue	<50
Xu et al. [123]	FOXD2-AS1^↑^	2018	China	106GC	1.75^∗^	1.04–2.97	Median	RT-PCR	Tissue	20–48

^a^One study involved GC and CRC. ∗ indicates adjusted HR; # indicates calculated HR of DFS and follow-up time; ! indicates studies included OS and DFS; ↑ or ↓ indicates upregulated or downregulated with poor prognosis. OS: overall survival; DFS: disease-free survival; HR: hazard ratio; CI: confidence interval; EC: esophageal cancer; GC: gastric cancer; CRC: colorectal cancer; GIC: gastrointestinal cancer; NR: no report; RT-PCR: reverse transcription PCR.

**Table 3:** lncRNAs and relevant targets in gastrointestinal cancer.

lncRNAs (*n* = 37)	Poor prognosis	Role	Relevant targets	Function	Reference
SNHG20^↑^	Upregulated	Oncogene	Cyclin A1, p21	Proliferation/invasion/migration	[29]
PVT1^↑^	Upregulated	Oncogene	EZH2, p15, p16, FOXM1	Proliferation/metastasis	[15, 34]
FEZF1-AS1^↑^	Upregulated	Oncogene	LSD1, P21, FEZF1	Proliferation/invasion/migration	[17, 19]
AGAP2-AS1^↑^	Upregulated	Oncogene	LSD1, EZH2, P21, E-cadherin	Proliferation/migration/invasion	[31]
XIST^↑^	Upregulated	Oncogene	miR-101, EZH2	Proliferation/migration/invasion/growth/metastasis	[32]
ATB^↑^	Upregulated	Oncogene	miR-200s, ZEB1, ZEB2	Invasion/EMT	[21]
UCA1^↑^	Upregulated	Oncogene	Ets-2, Sox4, miR-204, miR-204-5p, TGF*β*1	Migration/invasion/proliferation/apoptosis/chemoresistance/EMT	[64, 66, 96, 97]
NEAT1^↑^	Upregulated	Oncogene	Akt, vimentin, N-cadherin, Zo-1, E-cadherin	Proliferation/apoptosis/EMT/migration/invasion	[65, 73]
CCAT2^↑^	Upregulated	Oncogene	EZH2, E-cadherin, LATS2	Progression	[39]
CCAT1^↑^	Upregulated	Oncogene	c-Myc	Proliferation/migration/invasion	[43]
PANDAR^↑^	Upregulated	Oncogene	N-cadherin, vimentin, *β*-catenin, Snail, Twist, E-cadherin	EMT/growth/migration/invasion/apoptosis	[98]
H19^↑^	Upregulated	Oncogene	E-cadherin, Rb-E2F, CDK8, *β*-catenin, eIF4A3	Migration/invasion/proliferation	[48, 77, 84]
FOXCUT^↑^	Upregulated	Oncogene	FOXC1 (mRNA)	Proliferation/migration/invasion	[54]
MALAT1^↑^	Upregulated	Oncogene	EZH2, miR-218	Chemoresistance/EMT	[20]
uc002yug.2^↑^	Upregulated	Oncogene	RUNX1	Proliferation/migration/invasion	[25]
HOXA11-AS^↑^	Upregulated	Oncogene	EZH2, LSD1, miR-1297	Growth/migration/invasion/apoptosis	[16]
CCAL^↑^	Upregulated	Oncogene	AP-2α	Progression/multidrug resistance	[11]
GHET1^↑^	Upregulated	Oncogene	c-Myc (mRNA)	Proliferation	[18]
ROR^↑^	Upregulated	Oncogene	miR-145	Proliferation/migration/invasion	[69]
BC032469^↑^	Upregulated	Oncogene	miR-1207-5p	Proliferation	[71]
BLACAT1^↑^	Upregulated	Oncogene	EZH2, p15	Proliferation	[72]
GAPLINC^↑^	Upregulated	Oncogene	miR211-3p, CD44, PSF, NONO, SNAI2	Invasion	[12, 85]
SPRY4-IT1^↑^	Upregulated	Oncogene	Cyclin D1, MMP2, MMP9, E-cadherin, vimentin	Proliferation/migration/invasion/EMT/metastasis	[75, 88]
ZFAS1^↑^	Upregulated	Oncogene	EZH2, LSD1, CoREST, KLF2, NKD2	Proliferation	[76]
ANRIL^↑^	Upregulated	Oncogene	PRC2, miR-99a, miR-449a	Proliferation	[81]
LINC00152^↑^	Upregulated	Oncogene	EZH2, p15, p21	Proliferation	[83]
DUXAP8^↑^	Upregulated	Oncogene	EZH2, SUZ12, PLEKHO1	Proliferation/migration	[101]
SNHG15^↑^	Upregulated	Oncogene	MMP2, MMP9	Proliferation/migration/invasion	[104]
GAS5^↓^	Downregulated	Suppressor	E2F1, P21	Proliferation	[13]
lnc-GNAT1-1^↓^	Downregulated	Suppressor	RKIP-NF-*κ*B-Snail	Proliferation/migration/invasion/metastasis	[33]
FER1L4^↓^	Downregulated	Suppressor	miR-106a-5p	Proliferation/migration/invasion	[42]
MEG3^↓^	Downregulated	Suppressor	p53	Proliferation/apoptosis	[99]
MIR31HG^↓^	Downregulated	Suppressor	E2F1, P21	Proliferation	[45]
RP11-766N7.4^↓^	Downregulated	Suppressor	Vimentin, N-cadherin, E-cadherin	Migration/invasion/EMT	[26]
FENDRR^↓^	Downregulated	Suppressor	FN1, MMP2, MMP9	Migration/invasion	[94]
TUSC7^↓^	Downregulated	Suppressor	miR-211-3p	Proliferation	[100]
LINC00982^↓^	Downregulated	Suppressor	P15, P16	Proliferation	[103]
